# Visitation policies and practices in US ICUs

**DOI:** 10.1186/cc12677

**Published:** 2013-04-16

**Authors:** Vincent Liu, Julia Lindeman Read, Elizabeth Scruth, Eugene Cheng

**Affiliations:** 1 Kaiser Permanente Division of Research and Systems Research Initiative, 2000 Broadway (Webster Annex), Oakland, CA 94612, USA; 2 Kaiser Permanente Fremont Medical Center, 39400 Paseo Padre Parkway, Fremont, CA 94538, USA; 3 Kaiser Permanente Regional Quality and Regulatory Services, 1950 Franklin Street, Oakland, CA 94612, USA; 4 Kaiser Permanente San Jose Medical Center, 250 Hospital Parkway, San Jose, CA 95119, USA

## Abstract

**Introduction:**

Prior reports suggest that restrictive ICU visitation policies can negatively impact patients and their loved ones. However, visitation practices in US ICUs, and the hospital factors associated with them, are not well described.

**Methods:**

A telephone survey was made of ICUs, stratified by US region and hospital type (community, federal, or university), between 2008 and 2009. Hospital characteristics were self-reported and included the hospitals' bed number, critical care unit number, and presence of ICU leadership. Hospital and ICU visitation restrictions were based on five criteria: visiting hours; visit duration; number of visitors; age of visitors; and membership in the patient's immediate family. Hospitals or ICUs without restrictions had open visitation policies; those with any restriction had restrictive policies.

**Results:**

The study surveyed 606 hospitals in the Northeast (17.0%), Midwest (26.2%), South (36.6%), and West (20.1%) regions; most were community hospitals (*n *= 401, 66.2%). The mean hospital size was 239 ± 217 beds; the mean percentage of ICU beds was 11.6% ± 13.4%. Hospitals often had restrictive hospital (*n *= 463, 76.4%) and ICU (*n *= 543, 89.6%) visitation policies. Many ICUs had ≥ 3 restrictions (*n *= 375; 61.9%), most commonly related to visiting hours and visitor number or age. Nearly all ICUs allowed visitation exceptions (*n *= 474; 94.8%). ICUs with open policies were more common in hospitals with < 150 beds. Among restrictive ICUs, the bed size, hospital type, number of critical care units, and ICU leadership were not associated with the number of restrictions. On average, hospitals in the Midwest had the least restrictive policies, while those in the Northeast had the most restrictive.

**Conclusion:**

In 2008 the overwhelming majority of US ICUs in this study had restrictive visitation policies. Wide variability in visitation policies suggests that further study into the impact of ICU visitations on care and outcomes remains necessary to standardize practice.

## Introduction

Critical illness and intensive care have a profound and traumatic impact on the health and well-being of patients and their loved ones [1-3]. Previous reports suggest that many patients in the ICU are separated from their families and loved ones by widespread restrictive visitation policies that can negatively impact care and recovery [3-6]. However, limited data exist about the scope and variability of ICU visitation policies and practices across the United States as well as the hospital factors that influence them [7-10]. In this study, we conducted a survey of US ICUs to describe the current landscape of ICU visitation policies. We further aimed to evaluate whether key hospital characteristics were associated with visitation restrictions.

## Materials and methods

Based on the American Hospital Association 2008 Hospital Survey Database, we grouped hospitals as either university-affiliated hospitals, federal government (Veterans Health Administration) hospitals, or nonfederal and nonuniversity community hospitals. We aimed to survey all university and government hospitals with an ICU. We then stratified community hospitals (which make up the majority of US hospitals) based on their location in US regions (Midwest, Northeast, South, and West) and aimed to survey an equal percentage of eligible hospitals (25%) within each region to achieve a total of 670 surveyed hospitals (of an estimated 3,228 ICUs in the United States) [11].

For each hospital, we contacted the ICU leadership, if available, or ICU nursing staff familiar with visitation policies to conduct the telephone survey from 2008 to 2009. If a hospital's ICU personnel could not be identified or declined survey participation, the next hospital in the randomly generated sample by strata was surveyed. The 17-question survey ascertained hospital characteristics including each hospital's self-reported number of beds (total and ICU) and critical care units; if numbers were reported as a range (for example, 25 to 30 beds), we selected the mean value (28 beds). We calculated the percentage of critical care beds within each hospital (ICU bed percentage). We also ascertained the presence or absence of ICU leadership (medical director or clinical nurse specialist). Clinical nurse specialists typically have received training at the level of a master's degree and often take a lead role in staff education, protocol development, and standardizing nursing care based on current evidence.

We assessed visitation policies based on whether the hospital or ICU placed restrictions based on a total of five criteria: visiting hours; visit duration; number of visitors; age of visitors; and membership in the patient's immediate family. We designated hospitals with zero restrictions as having open visitation policies and those with any restriction as having a restricted policy. We also determined whether exceptions to the visitation policies were allowed within the ICU.

We described data as the number (frequency) and mean ± standard deviation. We used Spearman's correlation coefficient to assess the intra-hospital correlation between the number of hospital and ICU visitation restrictions. To determine the association between hospitals' ICU visitation policies and characteristics, we included key hospital characteristics as predictor variables in univariable and multivariable linear regression where the number of ICU restrictions was the outcome variable. Analyses were conducted using Stata/SE 11.2 (StataCorp. LP, College Station, TX, USA).

## Results

### Hospital characteristics

We contacted 695 hospitals; 87.2% (*n *= 606) completed the survey. Hospitals were located in 50 states and the District of Columbia. More than one-third were from the South (*n *= 222, 36.6%) and most were community hospitals (*n *= 401, 66.2%; Table 1). The mean hospital bed size was 239 ± 217 (median, 159). The mean ICU bed percentage was 11.6 ± 13.4%; the mean number of ICUs per hospital was 2.1 ± 1.8. A total of 62.2% of ICUs had a medical director and 39.0% had a clinical nurse specialist.

**Table 1:** Survey hospital characteristics

	Hospital region			
	
Variable	Northeast	Midwest	South	West
Number	103 (17.0)	159 (26.2)	222 (36.6)	122 (20.1)
Hospital type				
Community	55 (53.4)	102 (64.2)	154 (69.4)	90 (73.8)
Federal	26 (25.2)	31 (19.5)	40 (18.0)	18 (14.8)
University	22 (21.4)	26 (16.4)	28 (12.6)	14 (11.5)
Hospital bed number				
< 100	16 (15.5)	56 (35.2)	55 (24.8)	39 (32.0)
100 to 299	45 (43.7)	58 (36.5)	101 (45.5)	53 (43.4)
300 to 499	10 (9.7)	26 (16.4)	34 (15.3)	19 (15.6)
> 500	22 (21.4)	16 (10.1)	28 (12.6)	9 (7.4)
Not reported	10 (9.7)	3 (1.9)	4 (1.8)	2 (1.6)
ICU number	2.4 ± 2.1	2.0 ± 1.8	2.0 ± 1.6	2.0 ± 1.8
ICU bed number				
< 10	16 (15.5)	56 (35.2)	44 (19.8)	26 (21.3)
10 to 15	34 (33.0)	35 (22.0)	88 (39.6)	32 (26.2)
16 to 39	24 (23.3)	40 (25.2)	59 (26.6)	41 (33.6)
≥ 40	27 (26.2)	27 (17.0)	29 (13.1)	23 (18.9)
Not reported	2 (1.9)	1 (0.6)	2 (0.9)	0
ICU bed percentage	11.0 ± 9.9	12.0 ± 20.9	9.9 ± 7.1	15.2 ± 17.6
ICU clinical nurse specialist present	42 (40.8)	63 (39.9)	80 (36.0)	51 (41.8)
ICU medical director present	75 (73.5)	92 (58.6)	121 (54.8)	86 (71.1)

Data presented as number (percentage) or mean ± standard deviation.

**Figure 1:**
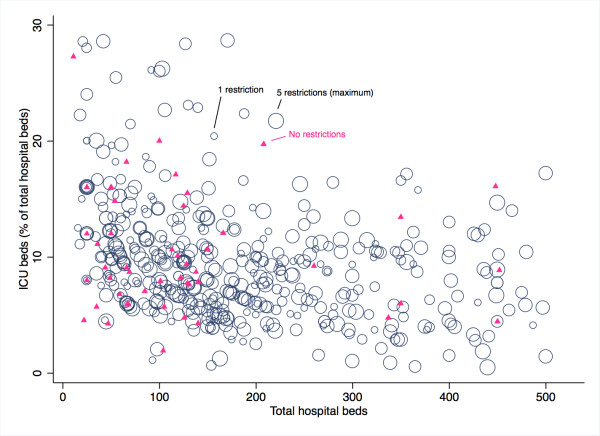
**ICU visiting policy restrictions based on number of hospital beds and percentage of ICU beds**. Weighted scatterplot of the number of ICU visiting policy restrictions based on the number of hospital beds and the percentage of ICU beds for hospitals with fewer than 500 beds. Pink triangles, hospitals that have no ICU visiting policy restrictions; Blue circles, size correlates with the number of ICU visiting policy restrictions (largest circle indicates a maximum of five visiting policy restrictions).

The majority of hospitals had restrictive hospital (*n *= 463, 76.4%) and ICU (*n *= 543, 89.6% visitation policies (Table 2). The mean numbers of restrictions were 1.4 ± 1.2 and 2.8 ± 1.5, respectively. Most ICUs had three or more restrictions (*n *= 375, 61.9%). The most common restrictions were related to visiting hours, followed by visitor number and age. Exceptions to the visitation policies were permitted in 94.8% of the ICUs. Within facilities, the correlation between the number of hospital and ICU visitation restrictions was moderate (correlation coefficient, 0.39). Figure 1 shows the distribution of ICU visitation restrictions based on hospital bed size and ICU bed percentage. Hospitals with fewer than 150 beds more frequently had open ICU visitation policies when compared with larger hospitals (16.8% vs. 5.1%; χ^2 ^*P *< 0.001).

**Table 2:** Survey hospital visiting hour policies, by hospital location

	Hospital location	
	
Category	Hospital-wide	ICU
Any restriction present	463 (76.4)	543 (89.6)
Number of restrictions (maximum of 5)	1.4 ± 1.2	2.8 ± 1.5
Restrictions present		
Visiting hours	448 (75.2)	487 (80.4)
Visit duration	42 (7.1)	239 (39.4)
Visitor number	134 (22.5)	408 (67.3)
Immediate family members	23 (3.9)	147 (24.3)
Based on visitor age	160 (26.9)	387 (63.9)
Allow exceptions to policy	-	474 (94.8)
Visiting hours	-	410 (82.8)
Visit duration	-	222 (44.9)
Visitor number	-	372 (75.2)
Immediate family members	-	132 (26.7)
Based on visitor age	-	332 (66.8)

Data presented as number (percentage) or mean ± standard deviation.

However, hospital bed size was not significantly associated with the number of ICU restrictions (Table 3); neither were hospital type, number of critical care units, or the presence of ICU leadership. Only the US region and ICU bed percentage were statistically significant in linear regression analysis; however, the effect size associated with ICU bed percentage was small (-0.03 for each percentage increase; 95% confidence interval, -0.05 to -0.002; *P *= 0.03). On average, hospitals in the Midwest had the least restrictive policies while those in the Northeast had the most restrictive.

**Table 3:** Variables associated with number of ICU visiting policy restrictions in univariable/multivariable linear regression models

	Point estimate from linear regression	
	
Variable	Univariable	Multivariable
Hospital size, per 100 beds	0.04 (-0.02 to 0.10)	0.01 (-0.10 to 0.11)
ICU bed percentage, per %	**-0.02 (-0.04 to -0.01)**	**-0.03 (-0.05 to 0.00)**
ICU number, per ICU	0.01 (-0.07 to 0.08)	-0.04 (-0.15 to 0.08)
Region		
Midwest (reference)	-	-
Northeast	**0.91 (0.52 to 1.30)**	**0.83 (0.43 to 1.23)**
South	**0.84 (0.53 to 1.15)**	**0.85 (0.53 to 1.17)**
West	**0.53 (0.17 to 0.90)**	**0.54 (0.18 to 0.91)**
Hospital type		
Community (reference)	-	-
Government	0.13 (-0.20 to 0.46)	0.16 (-0.17to 0.49)
University	0.08 (-0.29 to 0.46)	0.25 (-0.29 to 0.79)
ICU medical director present	0.18 (-0.08 to 0.44)	0.19 (-0.08 to 0.47)
Clinical nurse specialist present	0.06 (-0.20 to 0.32)	0.08 (-0.19 to 0.36)

Data in parentheses are 95% confidence interval. Bold data are statistically significant, *P *< 0.05. Multivariable linear regression model adjusted for all displayed variables.

## Discussion

In this survey of US hospitals, we found that their overwhelming default policy was to restrict ICU visitation. Among ICUs with restrictive policies, there was a high degree of variability in the number of restrictions and no significant association with hospital size or type, number of critical care wards, or leadership roles. The ICU policies were only moderately correlated with hospital-wide policies. In practice, however, nearly all ICUs allowed some exceptions to their visitation restrictions. Only a fraction of ICUs had open visitation policies and these were more common among small hospitals.

Critically ill patients often suffer from life-threatening disease and multisystem organ failure [12]. As a result, the modern ICU has evolved into a highly specialized unit designed to facilitate rapid diagnosis, continuous monitoring, and prompt delivery of multidisciplinary, multimodal, and technologically advanced therapies [13]. The results have been extraordinary, with patient survival steadily improving over time [14-16]. Because of the complexity of ICU care, prior small studies have raised concerns that open ICU visitation policies could harm patients by increasing physiologic stress, interfering with timely and safe care delivery, infringing on patient privacy, increasing exposure to infection, leading to caregiver exhaustion, and negatively impacting interactions with families [4,17-26].

Given these concerns, our finding that the majority of ICUs had restrictive and highly variable policies is not surprising. Prior studies have found similar results in US ICUs and international ICUs [6-10,21,26-28]. A survey among 171 hospitals in New England found that 32% had unrestricted visiting hours; however, most had restrictions on the age and number of visitors allowed [10]. Another survey of US ICUs, conducted by the American Association of Critical Care Nurses, also found high degrees of variability in visitation practice [9]. Giannini and colleagues reported that only a single Italian ICU, out of 257 surveyed, allowed open visitation [8]. No Belgian ICU, in a study by Spreen and Schuurmans, had an open visitation policy - defined as no restrictions on visiting hours, visit duration, and/or number of visitors [28]. Hunter and colleagues reported that 80% of ICUs in the United Kingdom imposed restricted visiting policies; they also noted wide variations in specific practices [27].

However, while historical practice among ICUs appears to have been to restrict visitation and we have seen concurrent substantial improvements in short-term mortality, new challenges in critical care have arisen. Survivors of critical illness and intensive care can suffer from post-intensive care syndrome - a condition whose sequelae include post-traumatic stress disorder as well as long-term physical and neurocognitive disability [1,2,29]. Furthermore, critical illness not only afflicts ICU patients, it also impacts patients' families [3,30-33]. Family members often struggle with decisions about their loved ones' end-of-life care and can, themselves, experience depression, anxiety, and post-traumatic stress disorder [3,30-35]. Visitation restrictions can thus further contribute to patients' and families' experiences of ICUs as disorienting places that enforce separation during challenging periods of critical illness and recovery [3,34-36].

As a result, numerous stakeholders and healthcare leaders have recommended liberalizing ICU visitation; Berwick and Kotagal declared restricted visiting practices as 'neither caring, compassionate, nor necessary' [3,5,37]. In 2010, US President Barack Obama also called on hospitals to foster open visitation policies [38]. Recent data suggest that open visitation policies do not adversely impact patient outcomes and represent only a moderate, and acceptable, intrusion on patient care [3,39-45]. Furthermore, family-centered care in the ICU is associated with improvements in the long-term psychiatric sequelae of critical illness, the trust between hospital staff and family members, and overall satisfaction with medical care [2,3,35]. Several studies also demonstrate the promise of interventions designed to provide families with a guided tour through the complexities of critical illness and to teach them how they can safely participate in ICU care [32,46-48].

Despite these reported benefits, we found that few ICUs had open visitation policies and they were more commonly located in small hospitals. Where ICUs had restrictive policies, we found wide variability in practice. Besides broad regional differences in ICU policies, other hospital characteristics were not strongly associated with the number of visitation restrictions. Instead, policies appeared to be distributed among hospitals without a clear pattern and probably reflect historical practices rather than evidence-driven decision-making [4]. Recent studies suggest that ICUs are actively rethinking their visitation policies to move towards more liberal visitation policies - a shift in the United States that has been supported by healthcare accreditation and regulatory agencies including the Joint Commission and the Center for Medicare & Medicaid Services among others [4,8,49].

Our findings should be interpreted in light of the study's limitations. First, the survey was conducted in 2008 and 2009. Given the increasing public awareness and unified sentiment that appear to favor open visitation policies since that time, a contemporary assessment of ICUs is urgently needed to determine whether policies have changed and what factors impact such changes. Second, while we sampled a large number of ICUs with high response rates, this survey represents the practices of fewer than 25% of US ICUs. Finally, additional factors that could influence ICU visitation policy (for example, the physical size of each ICU room, the presence of waiting rooms, the duration of visit times allowed) were not evaluated in this study and may offer additional insight into understanding the wide observed variability in practice across centers.

## Conclusion

The overwhelming majority of US ICUs in this study had restrictive visitation policies. While there were regional differences in ICU policies and open policies were common in smaller hospitals, other hospital characteristics were not strongly associated with the number of visitation restrictions. Wide variability in visitation policies suggests that further study into the impact of ICU visitations on patients and families are likely to influence and improve future practice.

## Key messages

• The majority of ICUs in the United States had restrictive visitation policies based on survey results from over 600 hospitals between 2008 and 2009.

• Hospitals in the Midwest region had the most liberal policies while smaller hospitals more frequently had open visitation policies.

• Hospital characteristics - including bed size, number of critical care units, presence of ICU leadership, and hospital type - were not associated with the number of visitation restrictions

• There was wide variability in ICU visitation policies and practices across a broad range of surveyed hospitals.

## Competing interests

The authors declare that they have no competing interests.

## Authors' contributions

VL had full access to all of the data in the study and takes responsibility for the integrity of the data and the accuracy of the data analysis - he participated in the conduct of the study; analysis and interpretation of the data; and preparation, review, and approval of the manuscript. JLR participated in the design and conduct of the study; collection, management, and interpretation of the data; and preparation, review, and approval of the manuscript. ES participated in the design and conduct of the study; collection, management, analysis, and interpretation of the data; and preparation, review, and approval of the manuscript. EC participated in the design and conduct of the study; collection, management, and interpretation of the data; and preparation, review, and approval of the manuscript. All authors read and approved the final manuscript for publication.
